# Supplementary material for: How Do We Define and Measure Optimal Care for Cancer Survivors? An Online Modified Reactive Delphi Study
Source: Cancers (Basel). 2021 May 11;13(10):2299. doi: 10.3390/cancers13102299 (PMC8150859; doi:10.3390/cancers13102299)
Supplement: Supplementary file 1 [file cancers-13-02299-s001.zip › cancers-1188003-supplementary.pdf]

Supplementary tables. Results of Round 1 survey

Table 1. Quality criteria added in the policy domain for R2 survey

**The organisation has a…**
policy that considers transitions in survivorship care (e.g. from paediatric to an adult care setting, acute to survivorship care)
policy around public reporting and dissemination of survivorship outcomes
policy that outlines the role of consumers in the design evaluation and reporting of survivorship programs

Table 2. Quality criteria added in the outcome domain for R2 survey

**The organisation has a process to…**
collect data on survivorship care providers' views of care
collect data on the number of survivors lost to follow-up
collect data on the number of referrals made for survivors

Table 3. Quality criteria modified in the policy domain for R2 survey

**Before**	**After**
**The organisation has a…**	
policy that documents survivorship care reporting requirements to a relevant hospital executive committee	policy that documents survivorship care reporting requirements to a relevant organisational executive committee
policy that documents survivorship care reporting requirements to a state government (e.g. Victorian Department of Health and Human Services)	policy that documents survivorship care reporting requirements to a government agency
policy for the collection of data on survivors' experiences of survivorship care (e.g. satisfaction with care, perceived timely access to care)	policy for the collection of data on survivors' experiences of survivorship care (e.g. satisfaction with care)
policy for the collection of data on carers' experiences of survivorship care (e.g. satisfaction with care, perceived timely access to care)	policy for the collection of data on carers' experiences of survivorship care (e.g. satisfaction with care)

Table 4. Quality criteria modified in the process domain for R2 survey

**Before**	**After**
**Cancer survivors are…**	
assessed for their risk of recurrent or new cancer, including family history	assessed for their risk of recurrent or new cancer, including family history (as necessary)
provided with referrals for genetic testing following primary treatment	provided with referrals for genetic testing (as necessary) following primary treatment
provided with surveillance recommendations for recurrent or new cancers	provided with recommendations regarding surveillance for recurrent or new cancers
provided with advice on medications to manage physical, psychosocial effects and/or chronic medical conditions	provided with advice on medications as appropriate to manage physical, psychosocial effects and/or chronic medical conditions
provided with advice on vaccinations (e.g. influenza)	provided access to advice on vaccinations (e.g. influenza)
provided with age- and gender-appropriate cancer screening or referrals to appropriate cancer screening services (e.g. mammograms)	provided access to age- and gender-appropriate cancer screening or referrals to appropriate cancer screening services (e.g. mammograms)
provided access to speciality care services (e.g. cardiology)	provided access to speciality care services to manage potential late effects, as necessary (e.g. cardiology)
provided access to access allied health services (e.g. nutrition, physical therapy, sexual health, rehabilitation)	provided access to access allied health services (e.g. nutrition, physical therapy, sexual health, rehabilitation, dental and podiatry services)
provided access to health information systems (e.g. telehealth programs)	provided access to telehealth services (e.g. video-based consultations)
provided access to remote surveillance programs where appropriate (e.g. remote monitoring)	provided access to remote surveillance programs where appropriate (i.e. having tests undertaken close to home, potentially without the need for clinical visits)
provided the opportunity to participate in funded research projects	provided the opportunity to participate in research projects including clinical trials

Table 5. Quality criteria modified in the outcome domain for R2 survey

**Before**	**After**
**The organisation has a process to…**	
collect data on survivors' return to work	collect data on survivors' return to previous functioning (e.g. work, study)
collect data on the number of survivors receiving recommended surveillance testing in the first year of follow-up	collect data on the number of survivors receiving guideline-compliant surveillance testing
collect data on the number of survivors who have a needs assessment following primary treatment	collect data on the number of survivors who have a needs assessment at certain time points post-treatment

Table 6. Quality criteria in the policy domain in order of importance (R1 survey)

**The organisation has a…**	***N***	**Mean rating**	**SD**	**% rated 4 or 5 on Likert scale**	**Result**
policy that requires the establishment or existence of a survivorship care program either on-site or by referral	64	4.47	0.70	91	**Included in R2**
policy that requires survivorship-focussed information to be available in other languages or in a different format for low literacy readers	63	4.47	0.85	90	**Included in R2**
policy for the evaluation of survivorship program and reporting of progress	62	4.34	0.81	89	**Included in R2**
policy for the provision of support services to survivors with special needs and from diverse cultural backgrounds (e.g. interpreters)	62	4.43	0.79	89	**Included in R2**
policy for the collection of data on survivors' experiences of survivorship care (e.g. satisfaction with care, perceived timely access to care)	61	4.44	0.70	87	**Included in R2**
policy that describes a framework for the provision of survivorship care	60	4.49	0.74	86	**Included in R2**
policy outlining the team of multidisciplinary health professionals included in the survivorship program	57	4.26	0.83	81	**Included in R2**
policy that describes the process of survivorship care reporting within an organisational reporting framework	56	4.24	0.85	80	**Included in R2**
policy on stratifying survivors to appropriate models of care	54	4.23	0.92	77	**Included in R2**
policy outlining the provision of needs assessment tools for survivors at certain time points post-treatment	53	4.06	0.90	76	**Included in R2**
policy that documents survivorship care reporting requirements to a relevant hospital executive committee	51	4.03	0.92	73	**Included in R2 after discussion**
policy for the collection of data on carers' experiences of survivorship care (e.g. satisfaction with care, perceived timely access to care)	51	4.01	0.96	73	**Included in R2 after discussion**
policy that describes how survivorship care data is collected and stored	49	3.96	0.95	70	**Removed after discussion**
policy that has a senior (executive) role identified as the organisation survivorship care champion	48	3.97	1.12	69	**Included in R2 after discussion**
policy that requires and records relevant staff training in survivorship care	46	3.96	0.95	66	**Removed after discussion**
policy that documents survivorship care reporting requirements to a state government (e.g. Victorian Department of Health and Human Services)	45	3.91	1.03	64	**Included in R2 after discussion**
policy that describes how survivorship care data is used for policy development and practice improvement	45	3.86	1.05	64	**Removed after discussion**

Table 7. Quality criteria in the process domain in order of importance (R1 survey)

Cancer survivors are…	*N*	Mean rating	SD	% rated 4 or 5 on Likert scale	Result
provided with surveillance recommendations for recurrent or new cancers	70	4.81	0.39	100	**Included in R2**
assessed for physical effects following primary treatment (e.g. pain, fatigue, weight loss or gain)	70	4.86	0.35	100	**Included in R2**
provided with treatment or referrals to manage physical effects of cancer and its treatment	70	4.80	0.40	100	**Included in R2**
provided with recommendations to reduce the risk of any physical effects (e.g. weight loss, exercise)	70	4.80	0.40	100	**Included in R2**
assessed for emotional and psychological effects of cancer and its treatment (e.g. anxiety, depression)	70	4.86	0.35	100	**Included in R2**
provided with treatment or referrals to manage psychosocial effects (e.g. to psychology services)	70	4.80	0.40	100	**Included in R2**
assessed for their risk of recurrent or new cancer, including family history	69	4.74	0.47	99	**Included in R2**
assessed for practical and social effects of cancer and its treatment (e.g. relationship difficulties, financial challenges, education and employment/return to work)	69	4.81	0.43	99	**Included in R2**
provided with care which is respectful of and consistent with their goals	68	4.76	0.49	97	**Included in R2**
provided access to a survivorship program which addresses the needs of cancer survivors either on-site or by referral	67	4.61	0.57	96	**Included in R2**
involved in care planning conversations and provided with a survivorship care plan	67	4.61	0.57	96	**Included in R2**
provided access to access allied health services (e.g. nutrition, physical therapy, sexual health, rehabilitation)	66	4.63	0.64	94	**Included in R2**
provided access to education and resources about the post-treatment phase which meets individuals' needs, understanding and health literacy	66	4.64	0.64	94	**Included in R2**
provided with a survivorship care plan that is shared with their primary care provider and/or other multidisciplinary health professionals involved in their care	65	4.59	0.67	93	**Included in R2**
stratified to appropriate models of care based on factors such as current needs and predicted risks	64	4.51	0.70	91	**Included in R2**
provided access to survivorship education and resources for their carers	64	4.39	0.69	91	**Included in R2**
provided with advice on medications to manage physical, psychosocial effects and/or chronic medical conditions	63	4.44	0.67	90	**Included in R2**

provided access to primary care services (e.g. GP visits and testing focused on management of chronic medical conditions, health promotion and disease prevention)	63	4.54	0.81	90	**Included in R2**
assessed for their self-management skills and appropriately stratified according to their ability to self-manage with support	62	4.40	0.77	89	**Included in R2**
provided access to health information systems (e.g. telehealth programs)	62	4.33	0.76	89	**Included in R2**
provided with age- and gender-appropriate cancer screening or referrals to appropriate cancer screening services (e.g. mammograms)	59	4.26	0.91	84	**Included in R2**
assessed for lifestyle behaviours with recommended management, or provided with an appropriate referral (e.g. quit smoking)	59	4.36	0.85	84	**Included in R2**
provided access to remote surveillance programs where appropriate (e.g. remote monitoring)	58	4.27	0.82	83	**Included in R2**
provided with advice on vaccinations (e.g. influenza)	55	4.04	0.98	79	**Included in R2**
assessed for adherence to recommended strategies to manage consequences of cancer and its treatment	54	4.07	0.77	77	**Included in R2**
provided with referrals for genetic testing following primary treatment	51	4.13	0.82	73	**Included in R2 after discussion**
provided access to speciality care services (e.g. cardiology)	51	3.97	1.05	73	**Included in R2 after discussion**
provided the opportunity to participate in funded research projects	51	3.99	0.97	73	**Included in R2 after discussion**
provided with support or referral for other medical or chronic conditions which are non-cancer related (e.g. diabetes)	50	4.01	0.96	71	**Included in R2 after discussion**
provided with access to screening services for exposure to infectious disease or conditions (e.g. Hepatitis B)	39	3.57	1.03	56	**Removed after discussion**

Table 8. Quality criteria in the outcome domain in order of importance (R1 survey)

The organisation has a process to…	*N*	Mean rating	SD	% rated 4 or 5 on Likert scale	Result
collect data on survivors' patient-reported outcomes	68	4.63	0.66	97	**Included in R2**
collect data on survivors' quality of life	68	4.63	0.54	97	**Included in R2**
collect data on survivors' patient-reported experiences of care	66	4.51	0.72	94	**Included in R2**
collect data on survivors' functional capacity	65	4.39	0.77	93	**Included in R2**
collect data on the number of survivors receiving recommended surveillance testing in the first year of follow-up	65	4.26	0.88	93	**Included in R2**
collect data on recurrence rates	62	4.43	0.84	89	**Included in R2**
collect data on the diagnosis of new cancers (for survivors)	60	4.36	0.87	86	**Included in R2**
collect data on the number of survivors provided with a survivorship care plan	60	4.29	0.74	86	**Included in R2**
collect data on survival rates (e.g. one and five-year survival rates)	59	4.41	0.89	84	**Included in R2**
collect data on survivors' hospital admissions	57	4.11	0.77	81	**Included in R2**
collect data on the number of primary care providers provided with a survivorship care plan	57	4.21	0.98	81	**Included in R2**
collect data on survivors' satisfaction with care	56	4.23	1.00	80	**Included in R2**
collect data on the number of survivors who have a needs assessment following primary treatment	56	4.14	0.94	80	**Included in R2**
collect data on the overall cost of survivorship care to the health service	55	4.16	1.06	79	**Included in R2**
collect data on carers' quality of life	54	4.16	0.81	77	**Included in R2**
collect data on the overall cost of care to survivors	54	4.19	0.97	77	**Included in R2**
collect data on the number of survivors stratified to different models of care (e.g. survivors self-managing their conditions)	53	4.11	0.89	76	**Included in R2**
collect data on survivors' return to work	51	3.96	0.88	73	**Included in R2 after discussion**
collect data on the average waiting time for follow-up services	50	3.96	0.95	71	**Removed after discussion**

collect data on the number of health professionals trained to provide survivorship care	48	3.91	1.00	69	**Included in R2 after discussion**
collect data on survivors' vaccination rates	32	3.40	0.97	46	**Removed**

Criteria: Discussion with the research team concerned criteria that had reached ≥50% but ≤75% agreement. Criteria included after discussion were based on available supporting literature.
